# New risks inadequately managed: the case of smart implants and medical device regulation

**DOI:** 10.1080/17579961.2015.1106107

**Published:** 2015

**Authors:** Shawn H.E. Harmon, Gill Haddow, Leah Gilman

**Affiliations:** a School of Law, University of Edinburgh, Scotland, UK; b School of Social and Political Science, University of Edinburgh, Scotland, UK

**Keywords:** risk, emerging technologies, medical devices, European Union, Implantable Smart Technologies Project

## Abstract

Many emerging technologies are associated with ‘risk’. While the concept of risk is protean, it is usually conceived of as the potential of something damaging or harmful happening. Thus, risks are a primary target of many regulatory regimes. In this article, after articulating an understanding of risk, we assess the European medical devices regulatory regime from a risk perspective, focusing on its handling of ‘smart’ implantable medical devices. In doing so, we discuss the empirical evidence obtained from expert participants in the Implantable Smart Technologies Project, which evidence is framed around three risk typologies: materiality, geography and modality. We conclude that none of these risks are sufficiently addressed within the existing regime, which falls down not just from a standards perspective, but also from the perspective of transparency and balance.

## 1 Introduction

All manner of risks to human well-being have been identified, including injury, invasive pathogens, genetic inheritance and the acquired and chronic diseases associated with aging, to name but a few. In an attempt to isolate and avoid these risks, and mitigate their deleterious consequences, we invent health technologies aimed at preventing, diagnosing and treating a wide range of conditions. Many of these health technologies are medical devices, a field which employs over 500,000 people in about 25,000 companies in Europe alone, and which generates annual sales of around €95 billion from some 500,000 products.^1^

The medical devices field is characterised by rapid and accelerating innovation, with drug development, tissue engineering, information and communication technologies and nanoscience playing a role in its evolution. As advances are made in materials, miniaturisation and energy generation, medical devices are becoming smaller, faster, more powerful and increasingly embedded or integrated into the body.^2^ Indeed, our lives are increasingly enacted within an intricate web of increasingly ‘smart’ technologies, which are not just performing *for* us, but also *on* us and *within* us. By ‘smart’, we mean they exhibit one or more of computational intelligence, autonomous operation and responsiveness to environmental changes (i.e. they monitor, transmit, and potentially initiate a treatment action).^3^

Given the above, there are very real concerns about the extent to which medical device regulations address the risks associated with these smart implanted medical devices (IMDs). In this paper, after unpacking the concept of risk, we discuss evidence generated in relation to risks, smart IMDs and the fitness of the European Union (EU) regulatory framework, which informs United Kingdom (UK) approaches to device testing and market authorisation.^4^ In the EU, IMDs are regulated by the Active Implantable Medical Devices Directive (AIMDD),^5^ the Medical Devices Directive (MDD)^6^ and the In Vitro Diagnostic Medical Devices Directive (IVDMDD).^7^ These have been amended by EU Directive 2007/47/EC.^8^ (References to the EU framework are to the consolidated version of the instruments.^9^) The UK implementing legislation is the Medical Devices Regulations 2002, as amended.^10^

The paper will unfold as follows. first, we introduce the empirical interdisciplinary project that informs this paper, highlighting some of the existing and emerging IMDs that are captured by the idea of smart IMDs. Second, we very briefly outline the EU/UK devices regime, which formed the backdrop against which our empirical evidence was taken. Third, we consider the risks that our respondents perceived as being particularly relevant to smart IMDs, although we emphasise that some of the concerns are common to all IMDs, not just smart IMDs. Fourth, we briefly discuss that evidence, considering what it says about the existing framework from risk and regulation perspectives. We conclude that smart IMDs have a number of features that translate into risks for which the existing framework is ill-equipped. Indeed, we argue that smart IMDs have features that challenge regulatory frameworks beyond the devices regime, and we make some modest suggestions for reform.

## 2 Risk and the implantable smart technologies project

Many emerging technologies, especially those that interact with the human body, are associated with ‘risk’. While there is significant sociological, medical, legal and economic literature on risk, there is no commonly accepted definition of risk, which has both descriptive and normative aspects.^11^ For present purposes, risk is conceived of as the potential for some negative or undesirable (i.e. destructive, damaging, harmful, unbalancing, undermining, affronting) state of affairs arising, with the implication that some action is warranted to avoid the risk, or to mitigate the consequences of it occurring, or both.^12^ Of course, this simple definition belies great complexity, for there is ample latitude for disagreement over:

- what counts as risk
- what should be acknowledged as proper variables in assessing/measuring risk
- what constitutes an acceptable level of risk
- what actions are most appropriate or effective for avoiding or mitigating risk.

The problems associated with risk are magnified (and multiplied) when the technologies that we devise to avoid, mitigate, or manage risk also *contribute* to the formation of *new* risks, which themselves might be individually or socially experienced.^13^

The ‘Implantable Smart Technologies Project’ (ISTP),^14^ was an empirical interdisciplinary project which had as one if its aims the exploration of ‘risk’ in relation to smart IMDs, a species of technology that might be viewed as giving rise to new and novel risks. At the outset, and drawing on literatures from the technical, social and legal disciplines, we conceived of risk as having two essential components, both common regulatory targets, namely ‘safety’ and ‘efficacy’. While safety is not an easy term to define, we take it to encompass the physiological and psycho-social harms that might result from receiving the IMD in question. Thus, safety measures are those actions which ameliorate the risk posed by the intervention itself. Efficacy, conversely, engages with that harm which the intervention is intended to avoid or mitigate. It highlights the fact that interventions are undertaken (and IMDs implanted) to serve a clinical purpose, and the question is therefore whether the intervention is delivering something effective; if the patient is not receiving what is expected in response to the condition, there can be profound health and well-being consequences (and so risks derived from non-efficacy).

Armed with this conception of risk, we undertook several empirical encounters to explore the term further (as well as the term ‘smart’). The first was a 2011 pilot workshop involving 13 experts from seven European jurisdictions and the USA undertaken by Harmon.^15^ The second was an independent allied project called Recovering Cancer Patients Views on in-Vivo Biosensors undertaken by Haddow.^16^ The third, undertaken more directly under the auspices of the ISTP, comprised 11 qualitative interviews with Edinburgh-based stakeholders.^17^ Interview respondents for this encounter were chosen for their involvement in the development, regulation, study or use of implantable technologies, and they were initially identified through existing networks of researchers, relevant university websites and local hospitals. The sample was expanded using a ‘snowball’ approach.^18^ Ultimately, five were professionals working at the legal/ethical interface of medical technologies, three were medical practitioners and researchers, one was a clinical scientist, one an engineer, and one an implanted patient, and each are described in Table 1.

All interviews were, with consent, recorded using a digital audio recorder and then transcribed verbatim. The transcripts were read multiple times by all members of the team, and analysis proceeded following a grounded theory approach,^19^ which is arguably now the standard form of qualitative analysis.^20^

Importantly, the ISTP was not concerned with scaffolding technologies such as hips, knees or other prosthetics. Rather, our investigations centred on more active or ‘smart’ implantable technologies: cochlear implants (CIs); implantable cardiac defibrillators (ICDs); in vivo biosensors (IVBSs) and deep brain stimulators (DBSs). These particular devices were chosen as the objects of the empirical element because: (1) they implicate very different physiological objectives and technical solutions all within the unifying field of IMDs; (2) they are either clinically available or close to clinical testing; (3) they have what we preliminarily considered to be ‘smart’ characteristics (i.e. they actively interacted, monitored and/or transmitted) and (4) they were familiar to our participant sample.

As can be gleaned from the more fulsome descriptions in Table 2, the subject IMDs are aimed at the diagnosis, monitoring or treatment of a disease or condition, and none of them operate by pharmacological, immunological or metabolic means. They rely on a power source not derivative of the body or gravity, and they are surgically or medically introduced into the body, and remain in the body post-procedure. As such, they are subject to the medical devices regime, which defines a ‘medical device’ as:^21^

any instrument, apparatus, appliance, software, material or other article, whether used alone or in combination, together with any accessories, including the software intended by its manufacturer to be used specifically for diagnostic and/or therapeutic purposes and necessary for its proper application, intended by the manufacturer to be used for human beings for the purpose of:

- diagnosis, prevention, monitoring, treatment or alleviation of disease,
- diagnosis, monitoring, treatment, alleviation of or compensation for an injury or handicap,
- investigation, replacement or modification of the anatomy or of a physiological process,
- control of conception, and which does not achieve its principal intended action in or on the human body by pharmacological, immunological or metabolic means, but which may be assisted in its function by such means.

An ‘active medical device’ is a device reliant on a source of electrical or other source of power other than that generated by the human body or gravity.^22^ An ‘active implantable medical device’ is an active medical device intended to be totally or partially introduced, surgically or medically, into the human body or natural orifice, and which is intended to remain after the procedure.^23^ (The new European regime would also capture these devices, as well as certain cosmetic devices, adopting harmonised definitions).^24^

Having outlined the project, and demonstrated that smart IMDs are caught by the regulatory framework, it is useful to offer a very brief overview of that framework before turning to the evidence on risks that was generated by our qualitative encounters.

## 3 Snapshot of the regulatory landscape

At the outset, we acknowledge that the EU is considering new legislation in this area,^25^ but the fact remains that the consolidated instruments identified above continue to operate, and will do so for the foreseeable future. Very generally, under the EU regime, regulatory functions are performed by national competent authorities.^26^ The UK’s national competent authority is the Medicines and Healthcare products Regulatory Agency (MHRA), which is tasked with ensuring compliance with standards, taking steps to prohibit or withdraw devices that do not meet standards, and recording, evaluating and reporting incidents.^27^ In practice, the MHRA approves, monitors, audits, and inspects ‘notified bodies’. There are six such bodies in the UK, and 76 across the EU.^28^

The notified bodies maintain a register of manufacturers, assess notifications of clinical investigations, enforce compliance of ‘CE’ marked devices (devices must generally bear the ‘CE’ marking, which signifies that it complies with quality standards),^29^ authorise the use of non-CE marked devices and undertake surveillance.^30^ In assessing a device, several factors are considered:

- the purpose and mode of action of the device;
- how long the device is intended to be in continuous use;
- whether the device is implanted, invasive or active;
- whether the device contains a medicinal substance that acts ancillary to the device.^31^

Based on these factors, devices are assigned to one of the following classifications:^32^

- Class I (non-invasive and low risk such as tables and wheelchairs);
- Class IIa (active and medium risk such as endoscopes and ultrasounds);
- Class IIb (active and used in critical conditions but still medium risk);
- Class III (invasive and high risk such as joints, stents, and ICDs).

The classification of a device determines the standards applicable for issuance of a CE mark, and the level of regulator involvement (and a device’s classification can change over time, shifting, for example, from Class II to Class III).^33^

## 4 Perceived risks of the subject in relation to IMDs

In this section, we explore the risks that the ISTP respondents considered to be of concern in relation to smart IMDs. They can be organised around three conceptual phenomena: ‘materiality’; ‘geography’ and ‘modality’.

### 4.1 Materiality

Materiality relates to the tangible physical material that is used to construct the IMD and its components. Given that all IMDs will invariably have some material element that will be of a different substance or nature to that of the receiving body, they will naturally pose some risk of physiological reaction in the recipient (and in this respect, smart IMDs are not particularly unique). R2-Engineer, reported:

[W]e know that the risks in [IMDs] are toxicity, potential radiation, and then rejection by the body, and possibly inflammation. These are the three known problem areas … If you had to make [an IVBS] out of aluminium, which the body really doesn’t like, then you’d have to think of some way of protecting the body from the aluminium by covering it up, sealing it off.

R3-Clinician-1 confirmed that some materials have good track records; gold and titanium, for example, are known to be inert in the body.

However, and arguably, risks associated with inflammatory or immunological responses to a device’s materiality might be accentuated in the case of smart IMDs because, as they become more sophisticated and perform more (and more complex) functions, they will become both smaller (and potentially more fragile) and more invasive (i.e. more deeply embedded and in parts of the body considered more integral or central to higher functioning and identity). R7-Clinician-3 recounted the problems that arose in relation to ICDs, which rely on narrow leads operating in a ‘hostile’ environment (i.e. the heart, which is constantly moving). Efforts to streamline ICDs resulted in the implantation of devices with leads that were too thin. Respondent-7-Clinican 3 went on to say:

[Companies] try and get a commercial edge by making features on their device … which make them attractive to us and to patients. So they’re trying to devise leads which are slimmer and easier to implant, and they devised this fidelis lead which was quite a small diameter lead, but it just wasn’t robust enough … I think it’s now accepted … that the leads are as small as they’re safely going to get …

In addition to reducing the performance of the IMD, which leaves the patient vulnerable to their disease/condition, chronic responses to IMD materiality can lead to full foreign-body reactions that ‘wall-off’ the device. This process, known as ‘bio-fouling’, can generate its own symptoms quite apart from the original condition that necessitated the device.^34^

The existing regulations stipulate that devices must be designed and manufactured in such a way as to remove or minimize as far as possible the risk of physical injury in connection with their physical or dimensional features,^35^ and that, during the manufacture of devices, particular attention must be paid to: 36

- the choice of materials used, particularly as regards toxicity aspects;
- mutual compatibility between the materials used and biological tissues, cells and body fluids, account being taken of the anticipated use of the device;
- compatibility of the devices with the substances they are intended to administer;
- the quality of the connections, particularly in respect of safety;
- the reliability of the source of energy;
- if appropriate, that they are leak-proof …

In short, both the responsibility for setting the risk tolerances and for managing the risks associated with materiality are placed on the device manufacturers. While this may be both practical and sensible, and while there is significant knowledge associated with materiality in the industry, our respondents expressed concerns about how this risk was approached under the existing regime. R9-Policymaker cautioned that bespoke clinical investigations around materiality (as well as other elements and functions of IMDs, both smart and non-smart) are often *not* undertaken. In fact, the ICD leads mentioned by R7-Clinician-3 were licensed and administered in the absence of human clinical data.^37^ Manufacturer duties were reiterated by R10-Government Researcher, who stated that ongoing monitoring of this risk was largely absent.^38^

Concerns around materiality become more critical when IMDs have a biological component. Work is underway to coat IMDs with polymers, medicines and sometimes tissue. It was agreed by our respondents that both biologics and nanomaterials introduce new levels of complexity and uncertainty because they naturally react and interact with the host’s body. However, the evidence base for how these materials interact is poor, and there is little regulatory assurance that good evidence will be generated.

A holistic view of our respondents’ evidence suggests that, for the subject smart IMDs and similarly complex emerging devices, the regulatory regime is insufficiently specific in the type and quality of evidence that would be required before a device is accepted for a specific classification and awarded a CE mark. This regulatory uncertainty, when combined with the scientific uncertainty often associated with new smart IMDs and some of the experimental materials, is undesirable, and potentially very harmful, and the matter will become more pressing as novel materials become more prevalent.

### 4.2 Geography

The second risk-related phenomenon, ‘geography’, refers to the location of the IMD within the body. This raises issues of invasiveness and identity. On the former, most smart IMDs require a surgical intervention whereby the device or a part thereof is implanted (although as they become smaller some can be injected). Sometimes, elements of the device (such as ICD and DBS leads) are run from one location within the body to another, sometimes through the veins. Obviously, the implantation process bears risks common to all surgeries, and this is addressed by other medical regulation. However, our respondents expressed the most concern in relation to IMDs implanted in the brain. It is already known, for example, that brain-implanted smart IMDs, in addition to prompting intense responses by patients and others, can impact on recipient identity and well-being (and there is significant literature on DBS affecting personality).^39^ On this issue, R2-Engineer stated:

I think I’d be, in principle, more concerned with something that was going inside my head than something going inside any other bit of me. Once you get south of the border, if the neck’s the border, I guess I would be pretty level about everything. There may be occasional bits and pieces that I might think about, but if it’s the body and not the thinking bit, I would say that’s one thing; if it’s the thinking bit, I would say that’s a different thing.

R5-Lawyer 2 concurred, stating:

There must be a sliding scale. If it’s just under your skin and in a kind of non-fetishized part of your body, a very boring bit of your body, then it’s nothing, it’s almost external. But when the organ in question becomes more about who you are and more, ‘woohoo’, it becomes spookier. I think that’s probably a spectrum from the wearable to the really banal implanted to the really special secret places where normally you would not go poking around.

R8-Bioethicist concurred, acknowledging that our understanding of the brain is so limited and so recent that IMDs associated with it raise special and widely agreed concerns about personality change, damage to thinking centres, external monitoring (i.e, brain-wave monitoring), and free will (i.e. ‘mind reading’ or control). In short, as explained by R1-Lawyer-1, perceptions of risk change dramatically once the IMD goes into the brain, for implantations in that organ are viewed as more invasive and more threatening to our capacities and identity.^40^

The regulation’s materiality provisions noted above would apply here, as well as more general provisions which state that the device’s use must not compromise the clinical condition or safety of the patient,^41^ and any side effects or undesirable conditions arising from the device must constitute ‘acceptable risks’ when weighed against the performances intended.^42^ Of course, the issue of what constitutes an acceptable risk remains undefined and reliant on *manufacturer* sensibilities, and the lack of understanding of certain organs and biological processes will continue to complicate smart IMD development aimed at certain interventions, leaving regulators with little independent knowledge on which to base decisions; it is the *manufacturer*, in cooperation with the notified body, which provides the evidence on which regulatory decisions are made, *and which determines the classi*fi*cation*. The national competent authority (the MHRA) only audits the process. However, different notified bodies have different standards, expectations for evidence and levels of competence, and manufacturers can choose which one to work with.^43^ Thus, manufacturers can choose notified bodies that are less demanding (i.e. that require the expenditure of less effort to meet regulatory standards). The result has been that many devices make it to market without rigorous supporting evidence (and certainly with less evidence than is expected of drugs).^44^

And there have been some very public failures in the context of non-smart IMDs. For example, in addition to the breast implants and metal-on-metal hip failures, it has been reported that a distal protection system for coronary artery interventions received an EU CE mark after a single study involving 22 subjects showed that it worked as intended, whereas approval was only granted in the US several years later after a randomised study involving 800 subjects.^45^ Indeed, many devices are granted the CE mark on the basis of literature alone so long as the manufacturer can demonstrate that the device is equivalent to one already on the market.^46^ The utility of this assessment is undermined when a device is approved based on a predicate device that was itself approved based on a predicate device.^47^

### 4.3 Modality

The third and final risk phenomenon that concerned our respondents relates to ‘modality’. This concept refers to the IMD’s method of action and functionality, and it has several elements – physiological responses, psychosocial responses, and third party access – each of which are addressed below.

#### 4.3.1 Physiological responses to smart IMDs

With respect to the physiological, it was felt that, as IMDs become more complex, they will interact in novel and unanticipated ways with the body, and have increased potential to malfunction, which could have profound consequences for recipients. R2-Engineer wondered about the repercussions of ‘misfiring’ treatments once IMDs are medicinally coated, or once drugs are put on chips for timed release or release in real-time response to physiological developments (both of which are innovation targets). The implications of concentrated doses of chemotherapy being released improperly into the body, for example, could be immense.

This is an issue of regulatory operation insofar as the framework already addresses the concerns voiced. Existing provisions, for example, state that devices must be designed and manufactured in such a way as to guarantee their safety characteristics and performances, with particular attention being paid to the proper functioning of the programming and control systems, taking into account the principles of development lifecycle, risk management, validation and verification.^48^ Other provisions state that, where devices incorporate a substance which, if used separately, may be considered a medicinal product, and which is liable to act upon the body with action ancillary to that of the device, the quality, safety and usefulness of the substance must be verified.^49^

The big question that remains, however, is whether the existing system is vigilant enough. It is generally considered not to be, with notified bodies – privately run bodies who are not public health agencies – seeing themselves as ‘clients’ of the manufacturers. They do not publish their decision-making process, nor the evidence provided by the manufacturer on which they base their decision, and they often keep information relating to safety confidential.^50^

#### 4.3.2 Psycho-social responses to smart IMDs

On the psycho-social element of modality, R5-Lawyer-2 emphasised that, while modality can have physical implications, it can also have strong psychological/emotional implications, particularly if the IMD interacts with (or indeed controls the operation of) an organ considered central to individual well-being. DBSs, for example, have elicited diverse and highly emotive responses. In particular, they have been known to cause significant personality change, such as hyper-mania, hyper-sexualisation and disinhibition.^51^ CIs can have profound life and socialisation implications,^52^ and have been the subject of heated debates around their perceived marginalisation of deaf culture and deaf identity,^53^ and the propriety of implanting them in children has been challenged.^54^

Despite smart IMD innovations almost invariably having psycho-social implications, the regulatory regime is silent on this issue. The difficulty, highlighted by R1-Lawyer-1, is that these symptoms and impacts will be largely unknown (possibly even un-theorised) until they present in users. The result is that this risk is characterised by a high degree of uncertainty. As such, robust *post-market surveillance and coordination* (i.e. effective monitoring in the clinical context) is critical, but our respondents unanimously thought that this was inadequate in the existing framework, from both a standards and practices perspective. In the UK, the following has been claimed:

The number of different types of devices on the market is about 80,000 in the UK and over 200,000 in Europe. Uncertainty surrounds the numbers because there is no publicly available list of devices being used … The MHRA does not know precisely which Class III devices (the most risky) have been cleared for use in the UK or Europe. … [T]he knowledge problem is compounded by the fact that NHS procedures are poorly coded … So while we can get detailed information about which drugs are being used in the NHS, the same does not apply to devices.^55^

Similar observations have been made elsewhere:

[A] researcher or regulator can readily identify all the drugs the person was taking before [a] procedure, but a detailed definition of the device itself is not routinely recorded in most clinical databases. Therefore, when the modified wiring of a pacemaker is found to cause potential fatal short circuits … it is hard to perform a systematic assessment, notification, or recall. Even car manufacturers have better databases to identify who uses their products …^56^

Moreover, the surveillance system is complaint-triggered rather than systematic, which means the data held by regulators is incomplete.^57^ Complaints are usually instigated by competitors, whistle-blowers within device manufacturers or subjects in clinical trials, as opposed to patients or their physicians.^58^ Manufacturers rarely actively collect post-market data,^59^ and they do not always publish recall data, nor identify which notified body issued the CE mark, or at what classification.^60^ More worrying is that, when manufacturers do issue safety notices (and the number of safety notices issued have increased dramatically, which is alarming in itself and reflects poorly on premarket assessments), regulators do not always respond with medical device alerts to the public.^61^

#### 4.3.3 Third party access to smart IMDs

The final aspect of modality relates to the inclusiveness of the ‘circle of control’,^62^ and this, more than any other issue, is unique to smart IMDs, and worried our respondents. This idea refers to the group of individuals who are implicated in the IMD’s ongoing operation. New and emerging smart IMDs are increasingly autonomous and reactive, often transmitting data to third parties for interpretation and subsequent action, either by that third party or by a clinician on advice from the third party. Given these functions, our respondents expressed concerns around decision-making (i.e. by designers and programmers) about how autonomous the devices will be and how secure they can be made. Linked to this is the issue of monitoring and adjusting how the devices operate within the body.

With ICDs, for example, the clinician is directly involved in programming the settings and sensitivities to physiological changes. For safety reasons, that programming is performed in hospital. R7-Clinician-3 reported:

The worry about having programmability remotely is that, if that programming goes wrong, you may not have a way to rescue the situation. So if you inadvertently programme something that is harmful to the patient, which occasionally happens because these are incredibly sophisticated devices, it’s not a good idea to have the person alone at home when that happens.

Several respondents expressed caution about expanding the circle of control beyond the treatment team, and they were reluctant to include patients themselves. R7-Clinician-3 explained:

[S]ome patients ask us to make alterations to their pacemaker which are clearly inappropriate. My worry is this balance between giving patients autonomy versus patient safety. Our job is to advise and facilitate treatments for patient safely, and in some ways you’d be passing a big part of the medical practitioner’s role over to the patient. I would question whether, with some patients at least … you [should] give your patient the power to make significant changes to the parameters of their pacemaker when that person isn’t medically trained and maybe doesn’t fully understand the effects on physiology of making certain adjustments …

Some IMDs have transmitters which will inform the hospital that they are active (e.g. ICDs can notify the hospital when they ‘go off’). This expands the circle of individuals who have knowledge about what the device is doing and when. Obviously, this has implications for patient privacy, which is eroded as more people become involved in treatment and device maintenance.

A more threatening aspect of privacy erosion that exercised our respondents was the risk that persons outwith the treatment team – and so bearing no duty of care to the patient – might hack into the device and gain knowledge, or, more alarmingly, alter its parameters. R7-Clinician-3 stated that, although ICDs can be interrogated online, they do not typically upload instructions remotely, largely because of these security concerns. Despite different levels of concern over hacking, R1-Lawyer-1 summed up many respondents’ fears:

[W]hat I would be interested in is the extent to which [the device] could be, or is, controlled remotely, for example. Or its ability to self-regulate, and in what ways, and if it is self-regulating, then what other controls could be exercised over it if it became inappropriate or unsafe.

Modality, and this element in particular, was the most concerning in part because our respondents viewed it as the least satisfactorily addressed by the regulatory framework. In particular, data transmission and performance modification grounded in the operation of software (i.e. security and decryption) is little addressed in the regulation.^63^ It has been reported that between 1999 and 2005 the number of recalls of software-bearing devices rose by more than 100%, and over 11% of the recalls related to software failures.^64^ Risks have resulted in harms from poor user interfaces, poor systems engineering and poorly articulated standards for safety, effectiveness, usability, dependability, security, none of which are adequately addressed in the existing regulatory regime.^65^ Additionally, the possibility of hacking into IMDs has already been demonstrated,^66^ and concerns have been expressed about the possibility of ‘remote homicide’ through device disruption.^67^

Some of the points of access to smart IMD software include device identification, data retrieval, device reconfiguration and software upgrading, multidevice coordination and communication, and manufacture audits in the event of failures, each of which could serve as a point of vulnerability.^68^ These combined with the reality of tens of millions of patients worldwide relying on software-driven devices for life-critical functions (and our respondents acknowledged that patient numbers will rise), means that the scope of this risk is immense, and the need for solutions to device security is pressing. Responses to issues such as software failure, data-flooding and necessity of upgrades must be both technology and regulation-based, but standards remain poorly specified, with no guidance being given to assessors (such as notified bodies) on appropriate and effective methods for evaluating how software performs its functions within devices and whether they are adequately secure,^69^ though such assessments are arguably mandated by the regulatory framework.^70^

Ultimately, while the list of device (and patient) vulnerabilities stemming from increased connectivity is growing, discourses about the appropriate balance between security, utility and traceability are underdeveloped.^71^ At base, it has been recommended that regulators should focus on outcomes rather than on standards, which will not prove durable or translatable across technologies (i.e. regulators should demand evidence of meaningful functional and clinical goals, and how they are met by the device).^72^ The importance of the software dimension to IMD regulation cannot be overstated, not only because of the potentially fatal physiological risks, but also the potentially profound psycho-social impacts on patients, who already may have anxiety around the IMD’s role in their body, fear about improper operation and stress relating to a desire to exercise more personal control over the device.

## 5 Discussion of the empirical evidence

Our empirical evidence relates to understandings of both the risks associated with IMDs generally, and smart IMDs more specifically, and the regulation that applies to them. The overall view of our respondents was that each of the above phenomena impact on ‘safety’ and ‘efficacy’, both of which ought to be well-defined and systematically tested for regulatory purposes. However, they also shared the broad view that more needs to be done to improve how smart IMDs are managed.

### 5.1 Risk evidence

On the issue of understandings of risk, the empirical evidence demonstrates that risks were associated with each characteristic (materiality, geography and modality). While many of the concerns were applicable to IMDs generally, some (e.g. accessibility under the modality phenomenon) were unique to new and emerging smart IMDs, and all were accentuated by smart IMDs. Risk concerns were driven in part by the invasiveness and novelty of the smart IMD in question, the former of which raises many well-known physiological risks and some less well understood psycho-social risks, and the latter of which introduces uncertainty about both risks and benefits. R3-Clinician-1 noted the following:

Some [IMDs], like a biosensor for drug delivery, wouldn’t yet be considered standard, so people might have more concerns around something they see as experimental … I think more established technologies would be fine, [but] others would need a little bit of work to get patient confidence and longer term safety data … [I]f you’re at the beginning of a technology then maybe you need a bit of confidence that it is going to be safe, but you don’t really know until 10, 15, 20 years have gone by.

R6-Clinician-2, R9-Policymaker, and R10-Government Researcher agreed, with the latter adding:

Some of [the risk] has to do with how experienced and effective clinicians become. So, whilst you would say [for a] pacemaker decades ago, ‘Oh, that’s very risky’, I wouldn’t see that as risky now. I see anything to do with the brain as being risky.

Ultimately, then, risk has a close relationship with novelty, and it is important to develop a reliable evidence-base so as to erode the uncertainty. It was agreed that better evidence of how new smart IMDs perform and how well they serve patients is needed. Developing this evidence-base, as noted by R1-Lawyer-1, is challenging, requiring cooperation and information exchange. When the implantation and/or operation of the device is invasive, the utility of information-sharing is increased. The same is true when the device contains features that might seem inherently risky, such as transmitting features.

### 5.2 Regulation evidence

Our respondents considered the regulatory framework to be wanting in relation to all three phenomena relevant to our risk components of ‘safety’ and ‘efficacy’. It was acknowledged that safety is addressed by the existing framework, but not sufficiently, and that efficacy is almost completely ignored by the regulation. In getting market approval, manufacturers need not demonstrate efficacy; the device must perform as intended by the manufacturer,^73^ and side effects must constitute ‘acceptable risks’.^74^ The need to demonstrate performance imposes a responsibility on manufacturers to demonstrate that the device operates as described, nothing more. The result has been that useless and dangerous devices could and almost certainly do receive market approval,^75^ which is particularly risky when one considers smart IMDs. A true efficacy requirement would demand evidence that the device actually delivers a beneficial clinical outcome (i.e. that it performs a valued and measurable function that offers a recognised benefit, and, where an existing device is in use, the new one delivers improved effectiveness). No such regulatory criterion is imposed.^76^ It has been observed that safety data is ‘hardly accessible to outsiders like patients’,^77^ and without information on how devices are functioning, clinicians have little basis on which to make treatment decisions, and patients have little information on which to base consent. The shortfalls in enforced processes to capture evidence has the consequence that decisionmaking might be questioned from a rationality perspective.^78^

Despite the prevailing ‘bottom-up’ and ‘responsive-mode’ approach of the EU being commended for delivering devices to the market quicker than a more top-down command-and-control system might,^79^ it has been heavily criticised for its complexity and lack of transparency,^80^ and that criticism was echoed by our respondents, who considered the framework lacking when measured against governance concepts viewed as important in the emerging technologies setting; in particular that of ‘transparency’. Mechanisms ensuring openness, information access (appropriate information to appropriate stakeholders) and clear lines of responsibility and liability should be defining features of the regulatory regime if safety and efficacy are to be achieved. At present, it was felt, they are not. In particular, the relationship between industry/innovators and regulators/monitors was felt by some respondents to be non-optimal, with serious barriers to assessing the level or extent of risks posed by devices, even recalled devices.^81^

## 6 Conclusion

Given the general acceptance that interventions on humans should be in the individual’s best interests, and should be evaluated continually for their safety, effectiveness, efficiency, accessibility and quality,^82^ one can conclude that the regulatory framework around smart IMDs is insufficient, with some glaring imbalances and gaps,^83^ and this was the general position of our respondents. With respect to imbalances, a holistic view suggests that ‘economics’ and ‘safety’, as opposed to ‘safety’ and ‘efficacy’, sit at the heart of the regime. The language of patient safety suffuses the instruments, which stipulate that IMDs must not compromise the clinical condition or safety of patients, or pose a risk to others, including those who implant them, and must comply with safety principles and the generally acknowledged state of the art.^84^ However, the more prominent ambitions appear to be market access and expansion.^85^ There is a clear push for early market availability of devices, as evidenced by the regulatory emphasis on manufacture, distribution, nomenclature and identifiers.^86^ The innovation-centric stance is underlined by the regime’s very early and prominent statement that members shall not create obstacles to devices entering the market or being made available to clinicians.^87^ Indeed, both EU and US regulators have been criticised for being ‘captured’ by industry, which is very much focused on markets and profit, and for allowing its safety assessments to be driven by politics and market ideology.^88^

With respect to gaps, the regulatory framework exhibits some critical lacunae, most particularly around generating evidence (pre and post-market) about both safety and efficacy. Given the proliferation, complexity and power of smart IMDs, and so the magnitude of their potential impact (both when operating properly and when malfunctioning), there is a pressing need to better address safety, and to more explicitly address efficacy. Concerns over unsafe materials which have already been used in non-smart IMDs underline the urgency for action on this front; IMDs are too often approved for use without sufficient understanding of their implications for health outcomes under the present regime, which permits devices to be marketed and implanted with dismayingly little understanding of key technical issues. And the lack of follow-on oversight offers unscrupulous manufacturers ample opportunities to take shortcuts (and, as our evidence highlights, shortcuts in relation to materiality, geography or modality can lead to considerable harm).^89^

The proposed amendments to the European framework are certainly a step in the right direction, but they are not enough.^90^ A regulatory regime which not only accommodates anticipated technological innovations and trajectories (and which captures aesthetic devices and implants), but which also articulates key guiding principles for both innovators and regulators, could profitably re-balance stakeholder positions. As IMDs become more complex, more embedded and more interactive, this will be important. Ultimately, a more robust and transparent regulatory framework, and one that takes grater notice of the patient, is warranted.^91^

## Figures and Tables

**Table 1:** ISTP Respondents

Reference	Respondent’s Description
R1-Lawyer-1	Experience assessing emerging technologies and policies
R2-Engineer	Experience innovating in the IMD field
R3-Clinician-1	Specialist in clinical oncology
R4-Clinical Scientist	Clinical physicist specialising in cochlear implants
R5-Lawyer-2	Experience assessing emerging technologies
R6-Clinician-2	Academic Cardiologist
R7-Clinician-3	Specialist in heart rhythm disorders
R8-Bioethicist	Served on research ethics and teaches medical ethics
R9-Policymaker	Civil servant and member of the MHRA
R10-Government Researcher	Senior civil servant with expertise in devices legislation
R11-Patient	Living with an Implantable Cardiac Defibrillator

**Table 2:** The Subject Implanted Devices

Device	Physical Description	Physiological Function
CI	Cochlear Implants are composed of an external component (a microphone, speech processor, and transmitter), which sits behind the ear, and an internal component (an electrode array), which is surgically placed within the ear.	Cochlear Implants can provide a sense of sound to those who are profoundly deaf or extremely hard-of-hearing. They do not restore ‘normal hearing’, but rather replace it by interacting with the environment and the auditory nerve to generate a physiological reaction.*
ICD	Implanted Cardiac Defibrillators are flat, metal devices containing programmable electronics and a battery. Though surgically implanted in the chest, they have leads that run to the heart.	Implanted Cardiac Defibrillators deliver electrical shocks to the heart when they sense the onset of life-threatening arrhythmias.
IVBS	In Vivo Biosensors are metal sensors, often coated in gold, that are extremely small, almost pinhead-sized, and contain an electrical power source.	In Vivo Biosensors measure a tumour’s biological environment, assessing whether real-time fluctuations in oxygen, Ph levels, etc., can be exploited to optimise the timing of treatment thereby overcoming radiotherapy resistance (i.e., treatment can be scheduled for when the tumour is least resistant).
DBS	Deep Brain Stimulators comprise a pulse generator implanted in the chest (near the collarbone), and subcutaneous leads running to electrodes implanted in the brain.**	Deep Brain Stimulators are intended to alleviate tremor, stiffness, and slowness caused by Parkinson’s. They are patient-controlled and there is some evidence that they may improve lung function, memory, and mood disorders such as depression.***

* Notes: For more on the development of this technology, see Raghu Garud and Michael Rappa, ‘A Socio-Cognitive Model of Technology Evolution: The Case of Cochlear Implants’ (1994) 5 *Organisation Science* 344.

** See <www.medicalnewstoday.com/articles/265445.php> (accessed 26 February 2014).

*** Michael Okun, Hubert Fernandez, Ramon Rodriguez and Kelly Foote, ‘Identifying Candidates for Deep Brain Stimulation in Parkinson’s Disease: The Role of the Primary Care Physician’ (2007) 62 *Geriatrics* 18; Walter Glannon, ‘Consent to Deep Brain Stimulation for Neurological and Psychiatric Disorders’ (2010) 21 *Journal Clinical Ethics* 104; Jonathan Hyam, ‘Controlling the Lungs Via the Brain: A Novel Neurosurgical Method to Improve Lung Function in Humans’ (2012) 70 *Neurosurgery* 469.
